# The intraoperative use of non-opioid adjuvant analgesic agents: a survey of anaesthetists in Australia and New Zealand

**DOI:** 10.1186/s12871-019-0857-9

**Published:** 2019-10-21

**Authors:** Venkatesan Thiruvenkatarajan, Richard Wood, Richard Watts, John Currie, Medhat Wahba, Roelof M. Van Wijk

**Affiliations:** 1 Department of Anaesthesia, The Queen Elizabeth Hospital, Woodville South, 5011 South Australia Australia; 2 The University of Adelaide, Adelaide, 5000 South Australia Australia; 3 Pain Management Unit, Flinders Medical Centre, Bedford Park, 5042 South Australia Australia

**Keywords:** Opioid analgesia, Non-opioid adjuvants, Opioid sparing, Intraoperative analgesia, Opioid survey

## Abstract

**Background:**

Opioids have long been the mainstay of drugs used for intra-operative analgesia. Due to their well-known short and long term side effects, the use of non-opioid analgesics has often been encouraged to decrease the dose of opioid required and minimise these side effects. The trends in using non-opioid adjuvants among Australian Anaesthetists have not been examined before. This study has attempted to determine the use of non-opioid analgesics as part of an opioid sparing practice among anaesthetists across Australia and New Zealand.

**Methods:**

A survey was distributed to 985 anaesthetists in Australia and New Zealand. The questions focused on frequency of use of different adjuvants and any reasons for not using individual agents. The agents surveyed were paracetamol, dexamethasone, non-steroidal anti-inflammatory agents (NSAIDs), tramadol, ketamine, anticonvulsants, intravenous lidocaine, systemic alpha 2 agonists, magnesium sulphate, and beta blockers. Descriptive statistics were used and data are expressed as a percentage of response for each drug.

**Results:**

The response rate was 33.4%. Paracetamol was the most frequently used; with 72% of the respondents describing frequent usage (defined as usage above 70% of the time); followed by parecoxib (42% reported frequent usage) and dexamethasone (35% reported frequent usage). Other adjuvants were used much less commonly, with anaesthetists reporting their frequent usage at less than 10%. The majority of respondents suggested that they would never consider dexmedetomidine, magnesium, esmolol, pregabalin or gabapentin. Perceived disincentives for the use of analgesic adjuvants varied. The main concerns were side effects, lack of evidence for benefit, and anaesthetists’ experience. The latter two were the major factors for magnesium, dexmedetomidine and esmolol.

**Conclusion:**

The uptake of tramadol, lidocaine and magnesium amongst respondents from anaesthetists in Australia and New Zealand was poor. Gabapentin, pregabalin, dexmedetomidine and esmolol use was relatively rare. Most anaesthetists need substantial evidence before introducing a non-opioid adjuvant into their routine practice. Future trials should focus on assessing the opioid sparing benefits and relative risk of using individual non-opioid adjuvants in the perioperative period for specific procedures and patient populations.

## Strengths and limitations

- This is the first survey across Australia and New Zealand of its kind
- The survey was anonymous, and examined diverse sample of anaesthetists in terms of location (public, private practices) and experience
- It included most of the available opioid adjuvants, and examined most of the obstacles for not using them
- A very low response rate of 33.4%, nonetheless, similar to recently published surveys from the ANZCA clinical trials network
- A response bias is possible as the sample is likely to contain practitioners with subspecialty interest. Regional and practice variations (e.g. tertiary vs rural practices, pain service availability) were not investigated in this survey

## Background

Opioids have always formed an integral component of a balanced anaesthetic, and remain the most effective drugs for the management of severe pain. Despite their advantages, they come with well-recognised adverse effects such as sedation, nausea and vomiting, constipation and respiratory depression [1–3]. Tolerance and hyperalgesia have been emphasized as adverse effects with longer-term (and occasionally short-term) use [1, 3]. In the community there has also been a general increase in opioid use with social as well as health implications (“the opioid epidemic”). Significant proportion of this epidemic is related to opioid overprescribing in the perioperative context and the anaesthetic implications of this has been discussed in the recent literature [4].

Multimodal analgesic regimens are commonly employed in the intraoperative period. Evidence shows that some adjuvants may enhance analgesic efficacy and facilitate opioid sparing with a reduction in opioid related side effects [3]. Non-opioid multimodal analgesia refers to paracetamol, non-steroidal anti-inflammatory drugs (NSAIDs), regional and local anaesthesia. Non-opioid Adjuvant drugs include N-Methyl-D-aspartate receptor (NMDA) receptor antagonists (e.g. ketamine, nitrous oxide), anticonvulsants (e.g. gabapentinoids), intravenous (IV) lidocaine, systemic alpha 2 agonists, magnesium sulphate, beta blockers, antidepressants (e.g. tricyclics, SNRIs). Their mechanism of action varies, and they act both centrally and peripherally, and the aim is to improve analgesia and reduce side effects [2].

Evidence supporting the use of these agents varies greatly, both with respect to the quality of evidence as well as the number of publications. Adjuvant usage appears to be influenced by patient, anaesthetic and procedure related factors, their availability, and the knowledge base and attitude of anaesthetists. In an earlier survey of anaesthetists, we carried out a cross-sectional questionnaire across the state of South Australia to assess the pattern of analgesic adjuncts used intraoperatively, to better understand their views and preferences [5]. After finding that the non-opioid adjuvants were sparingly used, we decided to survey anaesthetists across Australia and New Zealand to see if this was a pattern reflected across the two countries.

## Methods

The survey was approved by the Human Research Ethics Committee of the Central Adelaide Local Health Network (Reference: HREC/18/CALHN/183). The survey was pilot tested within our department (26 specialists), and the questionnaire was enhanced based on the feedback. The survey was reviewed by the Australian and New Zealand College of Anaesthetists (ANZCA) Clinical Trials Network Committee. An email link to the online survey was sent to 1000 randomly selected fellows out of the 5500 ANZCA fellows (specialist anaesthetists) in May 2018. The randomization was done by the ANZCA Clinical Trials Network Committee. The 1000 fellows were randomly extracted from the college’s database using a script. This is the standard practice adapted by our college for surveys. The survey was successfully delivered to 985 recipients (867 in Australia and 133 in New Zealand). A reminder email was delivered 2 weeks after the first email and the survey was closed after 4 weeks. The survey monkey (www.surveymonkey.com) platform was used for this anonymous survey. The IP addresses of the respondents were not collected.

The survey explored how frequently an individual agent was used for opioid sparing and the limitations in choosing an individual agent. The list included paracetamol, dexamethasone, NSAIDs, tramadol, NMDA receptor antagonists, anticonvulsants, IV lidocaine, systemic alpha 2 agonists, magnesium sulphate and beta blockers. This list was based on the most commonly used intraoperative agents in our institution, and agents which were previously examined in a cross sectional survey in South Australia [5]. The survey was not aimed at assessing the non-opioid sparing pharmacological properties of these agents.

The participants were questioned using two domains on each non-opioid adjuvant focusing on the frequency of use and any limitations as follows:

1. Frequency of use: a) never b) 10% usage c) 10–30% usage d) 30–50% usage e) 50–70% f) 70–90% g) 90–100%
2. Limiting factors in choosing a particular agent: a) time, b) cost, c) side effects, d) poor efficacy, e) lack of evidence, f) lack of experience with the drug, g) lack of knowledge about the agent, h) none, and i) other, with the option to free text.

The frequencies were chosen to reflect the usage as rarely (up to 30%), sometimes (30–50%), often (50–70%), very often (70–90%), almost always (90–100%).

A single reply was created for the frequency of use whereas multiple selections were allowed for the limitations. The respondents answered all questions.

Data were analysed using Microsoft Excel 2010. Descriptive statistics were used to present the practitioners demographic and practice characteristics. Data are expressed as a percentage of response for each drug. Percentages reported are based on actual numbers of respondents.

### Patient and public involvement

Since this survey was distributed to and was filled by Anaesthetists, there was no direct public or patient involvement in the survey.

## Results

Three hundred and twenty nine fellows responded to the survey yielding a response rate of 33.4%. Table 1 describes the demographic profile of the participants. Four out of five respondents were Australian, and this is approximately proportional to the numbers of fellows who were contacted. The majority of the respondents were experienced anaesthetists, with 58% (191) having more than 10 years post fellowship experience. There was also representative spread of private and public work, with just over half (53%) working in both sectors. To simplify the analysis, reported usage of an agent above 70% of the time was categorised as being “frequently used” and usage below 70% deemed as “less frequently” used.

**Table 1:** Demographics of the respondents. Figures are numbers (percentages) of respondents, *n* = 329

Characteristics	Frequency
Practice location	
Australia	271 (82%)
New Zealand	57 (17%)
Other	1
Specialist practice years	
< 5	63 (19%)
5–10	75 (22%)
> 10	191 (58%)
Practice type	
Public only	93 (28%)
Private only	60 (18%)
Public and private	174 (53%)

**Fig. 1:**
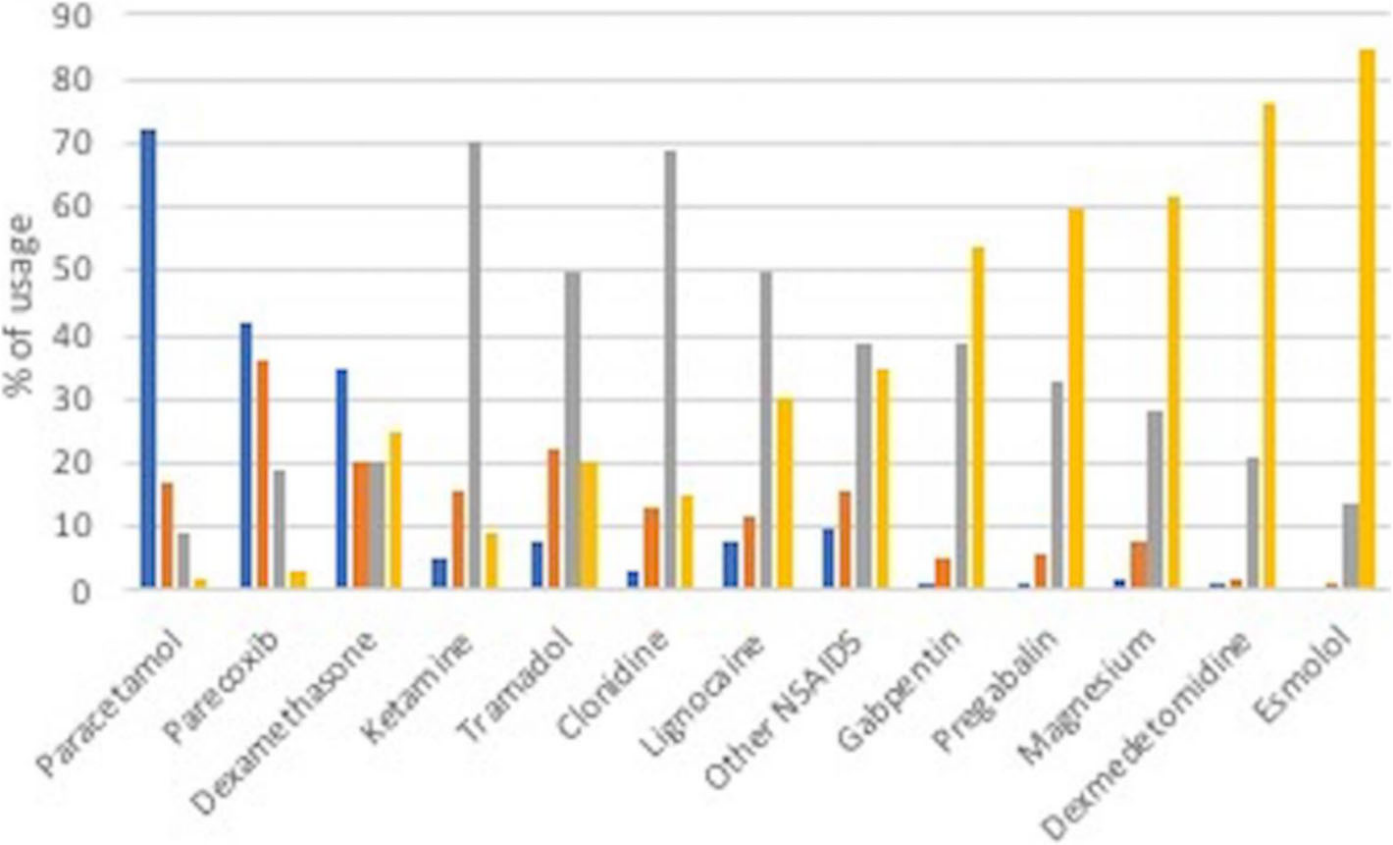
Use of opioid adjuvants reported as percentage of usage. Use ranked by frequency of administration. Blue: frequently used, usage above 70% of the time; orange: used 30–70% of the time; grey: used up to 30% of the time; yellow: never used. Values on the x-axis represent the proportion of usage of different agents and values on the y-axis represent percentage of responses for each category

Of all the agents, paracetamol was the most frequently used; 72% of the respondents reported frequent usage. This was followed by parecoxib (42% reported frequent usage) and dexamethasone (35% reported frequent usage). There was a steep decline in use of all the remaining adjuvants with less than 10% of anaesthetists reporting their frequent usage. The least used agents were dexmedetomidine, magnesium, esmolol, pregabalin and gabapentin; the vast majority of respondents suggested they would never consider these medications for their opiate sparing properties (Fig. 1).

Concerns which limited the use of individual agents varied and generally no one reason seemed to dominate for each agent.

Across the group, the main concerns were side effects, lack of evidence and experience. While side effect concerns dominated for tramadol, clonidine, ketamine, NSAIDs and gabapentinoids, lack of experience, and paucity of evidence of benefit dominated for magnesium, dexmedetomidine, and esmolol. Cost and time were of least concern to participants (Table 2).

**Table 2:** Leading limiting factors identified for the less frequently used opioid adjuvants, Values are percentages of actual responses

Agent	Predominant limiting factor
Tramadol	Side effects (80%)
Clonidine	Side effects (67%)
Ketamine	Side effects (61%)
Other NSAIDs	Side effects (57%)
Gabapentin	Side effects (41%)
Pregabalin	Side effects (37%)
Dexmedetomidine	Lack of experience (49%)
Magnesium	Lack of experience (31%)
Esmolol	Lack of evidence (29%)
Lidocaine	No specific factors identified (37%)

*NSAIDs* Non-steroidal anti-inflammatory agents

## Discussion

The survey provides a “snapshot” of the intraoperative use of non-opioid adjuvants across Australia and New Zealand. We found there were generally less non-opioid adjuvants used than in our earlier local survey across the state of South Australia [5]. Predictably, paracetamol and parecoxib topped the list of commonly used agents, as both have proven opioid sparing properties with a good safety margin. The results of this survey are reviewed below, with reference to the available published evidence. Paracetamol was the most frequently used agent by the respondents; 72% reported frequent use. It is an effective, well tolerated analgesic in the treatment of acute pain and all routes of administration have opioid sparing effects [3, 6, 7]. The convenience and safety of intravenous administration likely accounts for its widespread intraoperative use. A recent Cochrane review supports the safety and the clinical utility of IV paracetamol and pro-paracetamol in postoperative pain settings. However, it failed to reveal a clinically meaningful reduction in opioid-induced adverse events [8].

Parecoxib was the only intravenous selective Cox-2 inhibitor licensed in Australia at the time of this survey [9]. It is widely available in the operative environment and the dosing is convenient. Forty two percent the respondents reported frequent use, with side effects being the main limiting factor to its use. A recent systematic review and meta-analysis of randomized trials has shown that a combination of NSAIDs or Cox-2 inhibitors and paracetamol was superior to the later alone [10]. Based on the evidence and our survey findings, it is highly likely that the combination is often used in the perioperative setting. The prescription pattern of NSAIDs in a hospital setting is usually guided by the patients’ age as well as gastrointestinal and cardiovascular risk factors [11].

Dexamethasone delivers slight but clinically insignificant analgesic and opioid sparing effects; preoperative administration seems more effective than when given intraoperatively or postoperatively [3, 12]. However, it reduces nausea and vomiting, and improves recovery profile. While it was the third most preferred opioid sparing agent, it is possible that the respondents may have been using dexamethasone predominantly as an anti-emetic. The primary indication of utilising dexamethasone was not specifically asked in the survey, and this is acknowledged as a confounder. It is worth noting that dexamethasone is frequently used in conjunction with opioids in the setting of cancer pain [13].

The easy availability and favorable respiratory effects [3] makes tramadol an alternative to opioids in patients with sleep apnoea and in the bariatric population. Yet, its use was poorly reported in this survey, mainly because of potential side effects. This is in contrast to our earlier survey where more than half the respondents reported using it frequently [5]. When used as a single agent, it may be ineffective for moderate to severe acute pain [3].

There is mounting evidence that when administered in sub-anaesthetic doses, both IV and intramuscular ketamine decrease opioid consumption [14, 15]. Surprisingly, the acceptance of ketamine was also poor, in contrast to our earlier survey where almost half the respondents reported using it [5]. Ketamine has well established evidence as a perioperative analgesic and opioid sparing agent, but also has known adverse effects. Concerns about the occurrence of these (61.4% of respondents) might have limited its uptake into mainstream practice, despite that it is generally well tolerated in its analgesic dose range.

A reluctance in using IV lidocaine and magnesium was also observed. Though there is evidence supporting their role as non-opioid adjuvants, no specific limiting factor was reported for IV lidocaine by one-third of the respondents, whereas lack of experience was the foremost limiting factor reported for magnesium. IV lidocaine has proven opioid sparing effects and reduces pain intensity together with reducing the side effects of opioids (nausea and vomiting and ileus) [16, 17]. Perioperative IV lidocaine is particularly effective in abdominal surgery [18]. Indeed, perioperative infusions of lidocaine have been shown to have a preventative analgesic effect (effect lasting > 8 h after cessation of infusion) [19]. Lack of experience was the second major concern expressed in our survey in using lidocaine. Several Enhanced Recovery After Surgery (ERAS) society guidelines have incorporated IV lidocaine regimes; in place of intraperitoneal lidocaine for hysterectomy, and as a substitute to epidural for laparoscopic colorectal surgery [20]. On the other hand, a recent Cochrane review released in June 2018 has concluded that the beneficial effects of perioperative IV lidocaine on reduction of pain, ileus and nausea were uncertain due to limited quality of evidence [21].

Magnesium is an NMDA-receptor antagonist. It improves analgesia and has an opioid-sparing property when employed as an adjunct to IV morphine pain regimens, (meta-analyses and reviews [3, 22–24].). No serious adverse events were identified by the reviews which examined its role as an intraoperative adjunct [22–24]. Respondents’ disincentive for magnesium use did not dominate in any particular domain.

Systemic alpha-2 agonists were rarely used by survey respondents, with side effects being the main disincentive for clonidine use, and lack of experience with the use of dexmedetomidine. There is some evidence to suggest that their perioperative use may improve analgesia, reduce opioid consumption, and decrease nausea, without affecting the recovery times [3, 25]. Opioid sparing was reported across ten trials for clonidine and eight for dexmedetomidine [25]. On the other hand, a recent Cochrane review, whilst showing a slight opioid sparing effect in abdominal surgery, was unable to recommend this as a clinically significant finding [26].

Over half of the respondents reported that they have never used gabapentinoids, and the main reported concern was the side effect profile. Although better pain scores can be achieved with these agents, increased risk of dizziness, sedation, and respiratory depression (when given with opioids) were noted, with debatable significance of opioid sparing effect (NNT = 11 to reduce postoperative nausea and vomiting (PONV) with pregabalin) [27–29].

Not surprisingly, esmolol was one of the least preferred of all agents (85% of the respondents had never used it). Recent systematic reviews indicated an opioid sparing effect with esmolol in addition to improving pain intensity [30, 31]. It is worth noting that both these reviews include overlapping RCTs with significant heterogeneity and methodological deficiencies.

Our survey has several limitations. With a response rate of only 33.4%, a non-response bias is a definite possibility. We would have preferred a higher response rate. Regrettably, surveys take time to fill in, and we feel that one questionnaire and one follow up e-mail to a thousand anaesthetists keeps the balance between an acceptable sample size and not harassing our already busy colleagues. Our response rate is similar to recently published surveys from the ANZCA clinical trials network [32–34] and we believe that our results are likely to be representative and are worth reporting. As survey research is vulnerable in that it may deliver socially desirable answers, we have attempted to minimize this by maintaining respondent anonymity [35].

Choosing a non-opioid adjuvant is based on several patient, anaesthetic, and surgical factors such as the presence of neuropathic pain, chronic pain, opioid tolerance, bariatric surgery and sleep apnoea, to name but a few, and it is conceivable that surveying anaesthetists using precise opioid sparing scenarios, e.g.; bariatric surgery or the opioid tolerant patient may have generated different responses. However, it is likely that the respondents would normally care for a significant number of obese and opioid tolerant patients in their routine practice and this would be reflected in their survey responses. Another response bias is possible as the sample is likely to contain practitioners’ with subspecialty interest. Regional variations may not be represented in this survey. Also, similar to other surveys, it is likely that our survey would have captured “claimed” behaviour rather than actual behaviour. This survey did not include the use of regional anaesthesia techniques which now form a significant component of opioid sparing strategies, with some respondents alluding to this in their free text response. Further, the survey did not assess the correlation between the respondents age/work experience and the utilization of certain co-analgesics. Choosing a diverse sample in terms of location and experience as well as including most of the available opioid adjuvants were some of the strengths of our study.

The reasons for the reported low usage of non-opioid adjuvants in our study are likely to be multifactorial. Perceived lack of evidence was reported by significant proportion of respondents for agents such as lidocaine, gabapentinoids and magnesium. While this does not reflect the previously presented evidence for the utility of these agents, it may rather reflect the lack of transmission of evidence, and/or ‘evidence lag’ where there is a period of time before evidence is accepted into practice. It might have been useful if we included the question whether participants felt up-to-date with their knowledge on the topic. Perioperative medicine is increasingly protocol driven in an attempt to standardise practice and improve clinical outcomes. These protocols are normally part of enhanced recovery programs where there is growing evidence of the benefits of pharmacological and regional interventions to decrease opioid requirements [20]. As opioid sparing agents become part of these programs, we may well see an increase in their use in future years.

We feel that our survey has shown that there is a need for further high quality randomised controlled trials in the area of opioid sparing drugs; and specifically there is a need to address the question of whether the adverse effects of some opioid sparing medications are comparable or worse than those of the opioids themselves e.g. gabapentin, alpha 2 agonists, esmolol. Nonetheless, the survey also shows that despite adequate evidence for some adjuvants, the transmission of this evidence to practitioners and/or the translation of this evidence into practice, was still relatively low e.g. ketamine, NSAIDs and magnesium. Indeed a separate survey reports that opioids still constitute the mainstay for acute postoperative pain management in hospitalised patients, and that the need for effective analgesic medications with low adverse risk profile remains unmet [36].

We hope that this type of survey may encourage similar efforts in different geographic regions, and that pooled data regarding current practice and anaesthetists’ apprehensions can be used in designing future trials.

## Conclusion

This survey demonstrates respondent anaesthetists’ preferences and concerns in utilising non-opioid adjuvants for intraoperative opioid sparing across Australia and New Zealand. Most used paracetamol and parecoxib. A notable proportion routinely used dexamethasone though it is considered a weak agent commonly used for PONV. The uptake of tramadol, lidocaine and magnesium despite being supported by evidence was poor. Gabapentin, pregabalin, dexmedetomidine and esmolol use was relatively rare.

Our survey has provided an opportunity to review, and possibly improve, our opioid sparing practice, and given the low usage of some drugs, poses the question of whether there is any real appetite for change. Our results imply that opioids still constitute a major part of the intraoperative analgesic armamentarium. These findings are particularly important, and may indicate that the uptake of the current emerging trend towards “opioid free anaesthesia” would possibly require time. The survey also showed a potential lack of transmission of knowledge possibly implying a need for adequate ongoing education in this regard. Future trials should focus on assessing the clinical utility and the opioid sparing effects of using individual non-opioid adjuvants in the perioperative period for specific procedures and patient populations.

## Data Availability

The data that support the findings of this study have been attached as additional supporting files with the manuscript.

## Abbreviations

ANZCA: Australian and New Zealand College of Anaesthetist
Cox-2: Cyclooxygenase 2
ERAS: Enhanced Recovery After Surgery
IV: Intravenous
NMDA: N-Methyl-D-aspartate receptor
NSAIDs: Non-steroidal anti-inflammatory drugs
PONV: postoperative nausea and vomiting
RCTs: Randomised controlled trials
SNRI: Serotonin Noradrenaline Reuptake Inhibitors
